# Isolation, Identification, and Analyzing the Biological Characteristics of Pathogens Causing Stem Rot of Lanzhou Onion During Postharvest Storage and Studying the Influence of Pathogen Infection on the Active Components of Lanzhou Onion

**DOI:** 10.3390/jof10110789

**Published:** 2024-11-14

**Authors:** Ruoxing Wang, Hui Zhang, Qingru Zhang, Jihui Xi, Kunhao Jiang, Jinzhu Li, Huali Xue, Yang Bi

**Affiliations:** 1 College of Science, Gansu Agricultural University, Lanzhou 730070, China; wrx001011@163.com (R.W.);; 2 College of Food Science and Engineering, Gansu Agricultural University, Lanzhou 730070, China

**Keywords:** onion, postharvest storage, biological characteristic, active ingredients

## Abstract

This study was conducted in order to explore the pathogens that cause stem rot of fresh onions during postharvest storage, identify the incidence of stem rot, investigate the influence of pathogen infection on the active components of onion, and provide a theoretical basis for disease control during the postharvest storage of fresh onions. The pathogens were isolated and purified from the junction between the rotten and healthy tissues of onion stem rot that occurred naturally during storage at room temperature by tissue separation; then, the pathogens were identified by morphological and molecular biological techniques, the biological characteristics of the pathogens were analyzed, and finally, the influence of pathogen infection on the active ingredients of onion was studied. The results suggested that the main pathogens causing stem rot of fresh onions during postharvest storage were *Talaromyces pinophilus*, *Trichoderma simmonsii,* and *Talaromyces minioluteus*. The optimum colony growth conditions for *T. pinophilus* were as follows: a temperature of 30 °C, a pH of 7, light for 24 h, maltose as a carbon source, and peptone as a nitrogen source; the lethal temperature was 65 °C for 15 min. For *T. simmonsii*, the lethal temperature was 60 °C for 15 min, and the optimum sporulation conditions were a temperature of 25 °C, a pH of 5–7, light for 24 h, a carbon source of sucrose, and a nitrogen source of yeast powder. For *T. minioluteus*, the lethal condition was 65 °C for 15 min; the optimum colony growth conditions were a temperature of 25 °C, a pH of 8–9, 24 h of darkness, a carbon source of maltose, and a nitrogen source of peptone. The relative content of sulfur compounds, as the active components of onions, was much lower in the infected onions than in the healthy onions due to infection by the pathogens *T. pinophilus*, *T.simmonsii,* and *T.minioluteus*. This study will provide a theoretical basis for further effective control of the occurrence of postharvest stem rot diseases of onions.

## 1. Introduction

The onion (Allium cepa), a biennial herb of the Liliaceae family, is not only rich in nutrients but also possesses significant medicinal values, such as anti-cancer and antioxidant properties, earning the title “queen of vegetables” [1,2]. Onion is the second most widely grown vegetable globally, currently cultivated in over 170 countries [3]. China is among the top four producers of onions, along with India, the United States, and Japan. Onions are extensively cultivated across both the northern and southern regions of China, and the cultivation area continues to expand, making it one of the main vegetables in the country.

However, onions are often susceptible to infection by pathogenic fungi, leading to fungal diseases during both the field growth and storage stages. For instance, *Fusarium oxysporum* causes stem base rot, which can occur during both the growth and harvest stages of onions [4]. *Alternaria allium*, responsible for onion purple spot, primarily affects the leaves and pedicel during the growth stage and can occasionally damage the onion bulbs as well [5,6]. Onion downy mildew, caused by *Peronospora destructor*, predominantly occurs during the growth stage, targeting the onion leaves [7,8]. Most research on these diseases has focused on the growth stage of onions. However, onion stem rot is more prevalent and severe during the postharvest storage stage [9], which not only diminishes the market value of onions but also significantly reduces their storage and shelf life. The pathogens causing stem rot during postharvest storage vary significantly across different regions.

In addition, the infection of pathogenic microorganisms can impact the flavor and active component contents of host plants [10]. It has been reported that the main active components of onion bulbs are sulfides [11], including trisulfide and disulfide compounds [12,13]. Disulfide propane, in particular, has the highest sulfur content and is a key contributor to the distinctive flavor of onions [14]. The sulfides in onions have notable biological functions, such as lowering blood lipids, reducing blood glucose levels, and affecting hemolytic fiber. However, it has not been yet reported whether the invasion of pathogenic microorganisms influences the metabolism of sulfides in onion bulb tissue, potentially leading to a reduction in sulfide accumulation.

In this study, onion bulbs exhibiting typical symptoms of stem rot disease were collected from a farmers’ market in Anning District, Lanzhou, Gansu Province. Pathogens were isolated and purified from the tissue at the boundary between the diseased and healthy areas. The pathogens were identified using morphological and molecular biological techniques, and their pathogenicity was verified according to Koch’s postulates. Subsequently, the biological characteristics of the identified pathogens were analyzed, and the active ingredients of onions infected by the three pathogens were examined.

## 2. Materials and Methods

### 2.1. Samples

Onion bulb tissues displaying typical stem rot symptoms were collected from a farmers’ market in Anning District, Lanzhou City, during 2023–2024. The samples were placed into clean, sealed bags and transported back to the Chemical Biology Laboratory at the College of Science, Gansu Agricultural University, within 24 h. They were stored at room temperature (25 °C, 30% RH). Healthy onion bulb tissues, free of visible pests and diseases, were used as controls for the pathogenicity verification of the samples.

### 2.2. Methods

#### 2.2.1. Isolation and Purification of Isolates

Following the method of Xi [15], the bulb tissues with obvious disease symptoms were selected. Fragments (4 mm × 4 mm) were taken from the edge of diseased and healthy tissues and then disinfected in a 0.5% sodium hypochlorite solution for 5 min, followed by rinsing three times with sterile distilled water to remove any sodium hypochlorite residues. The fragments were then air-dried at room temperature and inoculated onto PDA medium. The PDA plates with the 4 mm × 4 mm fragments were incubated at 25 °C in the dark for 7 days. Colonies growing with different morphologies were separated by streak on new PDA medium, and this was repeated 4–5 times until a single colony was obtained. The experiment was repeated three times; one repeat included 5 colony cultures.

#### 2.2.2. Morphological Identification of Isolates

A 2 μL spore suspension of the isolated and purified pathogens was inoculated onto PDA medium and incubated at 25 °C for 7 days. Spore morphology was examined using a scanning electron microscope (ULTRAPLUS, ZEISS, Oberkochen, Germany), and spores were cultured on PDA medium with an improved glucose content. The morphology of the spore pedicles was observed under an optical microscope (CX21FS1C, OLYMPUS, Tokyo, Japan). A spore suspension (1 × 10^6^ spores/mL) was prepared, inoculated onto PDA medium, and cultured using the solid-insert method. Pathogens were identified based on their morphological characteristics by referring to the Fungal Identification Manual for *Talaromyces* spp. [16] and *Trichoderma* spp. [17].

#### 2.2.3. Molecular Biological Identification of Isolate

Mycelial DNA was extracted following the method described by Lv [18]. Fungal mycelium was collected from PDA medium and ground into a powder in the presence of liquid nitrogen. The powdered mycelium was then lyzed in platelet lysate, and a phenol–chloroform mixture was added. The mixture was thoroughly mixed and centrifuged, and the aqueous phase was transferred and mixed with 1 mL of ethanol solution. After thorough mixing and a second centrifugation, the supernatant was discarded. The precipitate was washed with ethanol solution, centrifuged again, and the supernatant was discarded. The precipitate was dissolved in 50–100 µL of an ethanol solution until fully dissolved. Finally, 2–5 µL of the DNA solution was used for electrophoresis on a 1% agarose gel for separation.

Based on the electrophoretic map, an appropriate amount of DNA solution was used as a template for subsequent PCR amplification. The PCR reaction system had a total volume of 50 μL, comprising 1 μL each of upstream and downstream primers, 2 μL of template DNA, and 46–47 μL of 1 × Taq PCR Mix. The primers used (ITS, Bt, RBP2, and CaM) and their sequences are listed in Table 1. Using the extracted isolate DNA as a template, the primers were designed according to the method of Xi [15]. The PCR amplification procedure was as follows: initial pre-denaturation at 94 °C for 5 min; denaturation at 94 °C for 10 s, annealing at 53 °C for 10 s, and extension at 72 °C for 30 s, repeated for 35 cycles; followed by a final extension at 72 °C for 5 min. The amplified product was then separated by electrophoresis on a 1% agarose gel.

The amplified fragments were sequenced by Beijing Bomad Biological Co., Ltd. (Beijing, China). The sequencing results were analyzed using NCBI BLAST (https://blast.ncbi.nlm.nih.gov/Blast.cgi, accessed on 13 April 2024), and homologous analysis was performed. MEGA 7.0 software was used to construct phylogenetic tree by adjacent method. The results of molecular biological identification and morphological identification were combined to determine the pathogen species.

#### 2.2.4. Pathogenicity Testing of Isolates

Onion bulb tissues without visible signs of diseases or pests were collected and disinfected with 0.1% sodium hypochlorite for 15 min, followed by rinsing three times with sterile water to remove any sodium hypochlorite residues. Inoculation holes (1 mm in diameter and 3 mm deep) were made in the onion bulbs using a sterilized pipette tip. Spore suspensions (1 × 10^6^ CFU/mL) of the three isolated and purified pathogens were prepared, and 10 μL of each spore suspension was separately injected into the onion bulbs, with sterile water serving as control. After air drying, the inoculated onion bulbs were incubated in the dark at 20 °C and 50% RH. Samples were taken every 7 days to calculate the disease incidence and disease index. The disease incidence and disease index were calculated as follows:

Disease index = ∑ (number of disease grade × representative value of each disease grade)/(total number of plants × representative value of highest disease grade) × 100%

Disease incidence = number of diseased plants/total number of plants × 100%

Four groups of treatments were inoculated with the three major pathogenic strains, and the control group was kept uninfected, with each treatment repeated three times (each repeat consisted of 10 pieces of healthy onion). In the course of our experimental protocol, one variant entailed the utilization of ten onions, each of which was required to undergo a triplicate parallel processing. Across four separate treatments, this cumulatively resulted in the processing of a total of 120 (10 × 3 × 4 = 120) samples. After 21 days of incubation, isolates were collected and purified from the junction between the rotten and healthy tissues and were compared with the originally inoculated pathogens. Isolates that conformed to Koch’s postulates [15] were selected for further study. The disease severity was graded on a scale of 0 to 4, as described in Table 2.

#### 2.2.5. Effects of Temperature, Light, pH, Carbon and Nitrogen Sources and Different Humidity Conditions on the Growth and Sporulation of Pathogens

A 2 μL spore suspension was inoculated at the center of PDA medium and incubated in the dark at temperatures of 15, 20, 25, 30, and 35 °C for 7 days. The colony morphology was observed, and the colony diameter was measured using the cross method. The identified pathogens were replicated three times in each temperature condition. Spore production was measured and calculated using a hemocytometer (blood cell counting plate) method. Plate with the identified pathogen was replicated three times to determine fungal sporulation.

A 2 μL spore suspension with a concentration of 1 × 10^6^ CFU/mL was inoculated at the center of PDA medium under three different conditions: 12 h of light alternating with 12 h of darkness, 24 h of continuous darkness, and 24 h of continuous light for 7 days. The colonies were observed, and their colony diameters were measured using the cross method. Spore production was determined using the hemocytometer method. Plate with the identified pathogen was replicated three times to determine fungal sporulation.

The pH of the PDA medium was adjusted using HCl and NaOH solutions to create pH levels of 4, 5, 6, 7, 8, 9, 10, and 11. A 2 μL spore suspension with a concentration of 1 × 10^6^ CFU/mL was inoculated at the center of each PDA plate. Colony diameters were measured using the cross method, and spore production was determined using a hemocytometer. The identified pathogens were replicated three times in each pH level.

Czapek medium was prepared by dissolving 3 g NaNO_3_, 1 g K_2_HPO_4_, 0.5 g MgSO_4_·7H_2_O, 0.5 g KCl, 0.01 g FeSO_4_·7H_2_O, 30 g sucrose, and 20 g agar in 1000 mL of sterile water. This medium was used to investigate the sources of carbon and nitrogen. The nitrogen source was maintained as NaNO_3_, while sucrose was replaced with equivalent carbon masses of glucose (31.58 g), maltose (30 g), cyclodextrin (28.42 g), mannitol (31.93 g), or fructose (31.58 g) as the carbon source. For nitrogen sources, ammonium sulfate (2.33 g), yeast extract powder (6.16 g), peptone (0.22 g), glycine (2.65 g), and urea (1.06 g) were used in place of sodium nitrate. Czapek medium served as the blank control.

The 2 μL spore suspensions of the three pathogens were separately cultured at a constant temperature for 7 days in Czapek medium with different carbon sources. Colony growth was observed, and colony diameters were measured using the cross method. Spore production was measured using a hemocytometer. Plate with the identified pathogen was replicated three times to determine fungal sporulation.

Colonies were cultured in Petri dishes using the slide method [24]. The relative humidity was adjusted to 23 ± 2.0%, 33 ± 2.0%, 43 ± 2.0%, 59 ± 2.0%, 65 ± 2.0%, 75 ± 2.0%, 84 ± 2.0%, and 93 ± 2.0% using saturated solutions of CH_3_COOK, MgCl_2_, K_2_CO_3_, NaBr, CoCl_2_, KNO_3_, KCl, and NaCl, respectively. Spore production and spore germination rates were calculated after 12 h of incubation at different relative humidity levels under dark, constant temperature conditions. Spore germination under these conditions was observed using an optical microscope. Plate with the identified pathogen was replicated three times to determine fungal sporulation.

#### 2.2.6. Analysis of Onion Active Components

##### Sample Preparation

Healthy onion bulbs without visible mechanical damage or pests were selected as the control group, while onion bulbs inoculated with the three pathogens were designated as the treatment groups (WRX-1, WRX-2, and WRX-3). For the treatment groups, 30 g of tissue was collected from the rotten areas, and for the control group, 30 g of tissue was collected from healthy areas. The samples were then quickly ground into powder using liquid nitrogen and stored at −4 °C for later use.

Following the method of Fernandes [25], 10 g of sample powder was accurately weighed and transferred into a headspace vial. Then, 5 mL of saturated NaCl solution was added, and the mixture was heated and stirred at 1600 r/min for 20 min to equilibrate the volatile substances in the vial. After equilibration, the pre-conditioned extraction fiber was inserted into the headspace vial, the fiber head was extended, and volatiles were absorbed at a specified temperature for a set duration. The fiber head was then retracted, and the extraction head was quickly inserted into the gas chromatograph inlet. The fiber head was extended and analyzed at 250 °C for a specified time. Data collection by the instrument commenced as the fiber head was inserted. The fiber used for extraction was a 50/30 μm DVB/CAR/PDMS fiber, and the extraction was performed at 60 °C for 60 min. The experiment was repeated three times.

##### GC–MS Condition

GC conditions: The column is a capillary column of HP-INNOWAX model, and the specification is 60 m × 0.250 mm × 0.5 μm. The sample inlet temperature of the gas chromatograph is 250 °C. The programmed temperature condition is as follows: the initial temperature is 60 °C, hold for 1 min; the temperature is raised to 180 at the rate of 2 °C/min; when the temperature rises to 230 °C, hold for 5 min. The carrier gas is high-purity helium (purity ≥ 99.999%); column flow rate is 0.98 mL/min; and shitter ratio is 20:1.

MS condition: electron bombardment ion source; electron energy, 70 eV; transmission line temperature, 230 °C; ion source temperature, 230 °C; scanning mode: Full Scan; ion mass scanning range m/z, 25~500 u.

#### 2.2.7. Statistical Analysis

Data (mean and standard deviation) were calculated using Excel 2019; SPSS 21.0 was used to analyze the significance of differences (*p* < 0.05), and all data in this study were created using Origin 2020 (Northampton, MA, USA).

## 3. Results

### 3.1. Symptoms of Onion Stem Rot During Postharvest Storage Stage

**Figure 1:**
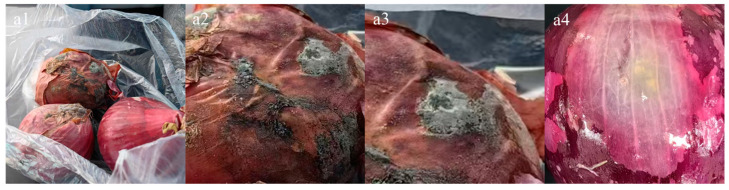
Symptoms of onion stem rot during storage stage. (**a1**) symptoms of onion stem rot (**a2**) local and special symptoms of onion stem rot. (**a3**) magnification of local and special symptoms of onion stem rot. (**a4**) water stains, a disease symptom of the tissue under the mold layer of stem rot.

The onion bulbs with typical stem rot symptoms were collected from the farmer’s market in Anning District, Lanzhou City, Gansu Province of China; the disease symptoms are clearly visible (Figure 1). The onion surface tissue is scattered with gray–green spots and flocculent mold spores and had a certain pungent odor. The tissue under the mold layer is wrinkled, soft, light in color, and accompanied by water stains.

### 3.2. Identification of the Pathogen Causing Onion Stem Rot During Storage

#### 3.2.1. Morphological Identification

**Figure 2:**
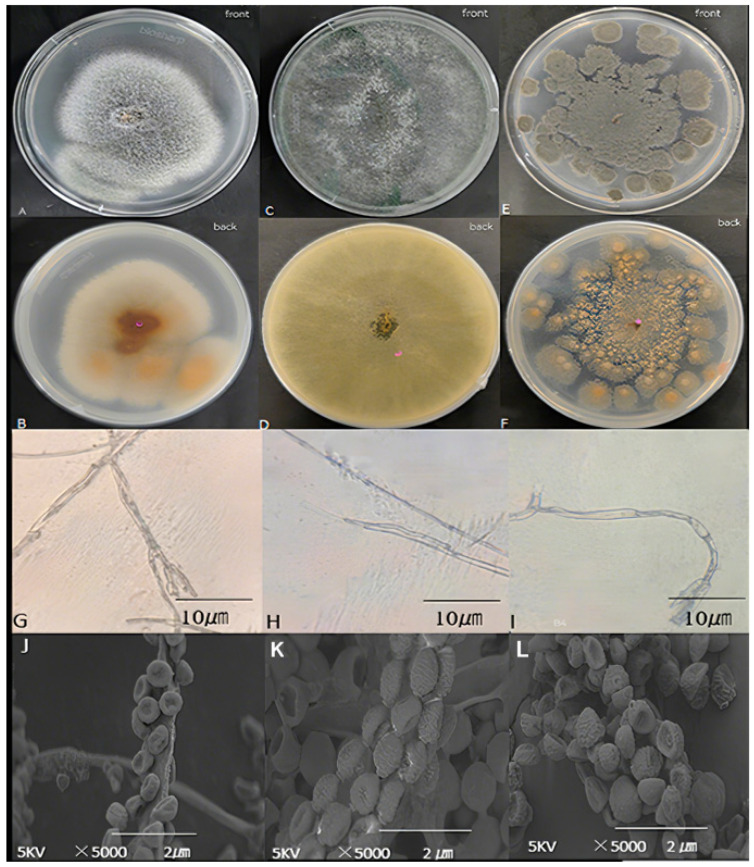
Morphological observation of pathogens. (**A**,**B**) WRX-1 colony morphology; (**C**,**D**) WRX-2 colony morphology; (**E**,**F**) WRX-3 colony morphology; (**G**) WRX-1 spore morphology; (**H**) WRX-2 spore morphology; (**I**) WRX-3 spore morphology; (**J**) WRX-1 sporophyte morphology; (**K**) WRX-2 sporophyte morphology; (**L**) WRX-3 sporophyte morphology.

Three strains were isolated and purified from the tissues showing typical symptoms of stem rot in fresh onions. The colony morphology, spore morphology, and spore stalk morphology were observed. The WRX-1 colony is grayish green on the front side of the PDA medium (Figure 2A) and orange on the back side (Figure 2B), with radial folds and fluffy texture. The conidia are erect with green branches, and the top branches are broomlike (Figure 2G). The conidia are loosely clustered on the pedospora. The achromatic monospore is round or oblate (Figure 2J). The surface of the monospores has particles (2–3.5) μm × (100–160) μm in size. Referring to the fungal identification manual [16], the isolate of WRX-1 was preliminarily identified as *Talaromyces pinophilus* (Figure 2).

The WRX-2 colonies on the PDA medium are gray–green on the front side (Figure 2C) and yellow on the back side (Figure 2D). The conidia are erect, unbranched, green, with clamp-like tips (Figure 2H). The conidia are compactly clustered on the pedospora. The achromospora are round (Figure 2K); the surface particles are not smooth, and their size is (2~3.5) μm × (100~160) μm. Based on the fungal identification manual [17], the isolate of WRX-2 was preliminarily identified as *Trichoderma simmonsii* (Figure 2).

The WRX-3 colony appears grey–green on the front of the PDA medium (Figure 2E) and is surrounded by a white fuzz. The back is orange–yellow (Figure 2F). The colonies appear as scattered spheroids. The spore peduncle is curved, branching, and green; the apex branch is hand-shaped (Figure 2I). The conidia are clustered tightly on the spore stem. The achromatic monospore is spherical with a concave center (Figure 2I). The surface is corrugated. The size of the conidia is (2~3.5) μm × (100~160) μm. Combining with the fungal identification manual [16], the isolate of WRX-3 was preliminarily identified as *Talaromyces minioluteus* (Figure 2).

#### 3.2.2. Molecular Biological Identification

**Figure 3:**
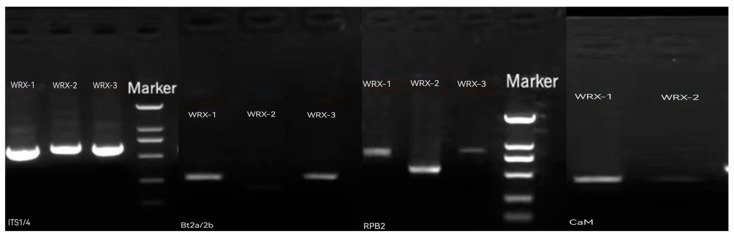
Gel electrophoresis of PCR amplification products.

The three strains were amplified by PCR using the *ITS*, *BT*, *CaM*, and *RPB2* primers. The amplification products were analyzed by 1% agarose gel electrophoresis. The resulting gel electrophoresis diagram is shown in Figure 3. The sequence sizes of the three strains amplified by the *ITS* primers were 574 bp (WRX-1), 611 bp (WRX-2), and 599 bp (WRX-3). The sequence sizes of the three strains amplified by the *BT* primers were 464 bp (WRX-1), 362 bp (WRX-2), and 473 bp (WRX-3). The sequence sizes of the three strains amplified by the *RPB2* primer were 840 bp (WRX-1), 537 bp (WRX-2), and 841 bp (WRX-3). Additionally, the sequence sizes of the two pathogens amplified by the CaM primer were 667 bp (WRX-2) and 655 bp (WRX-3).

**Figure 4:**
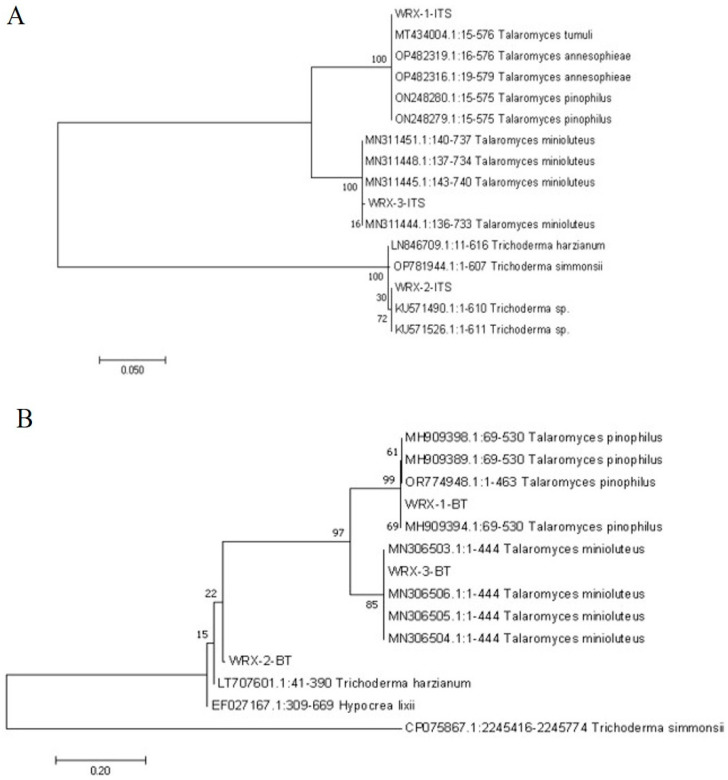

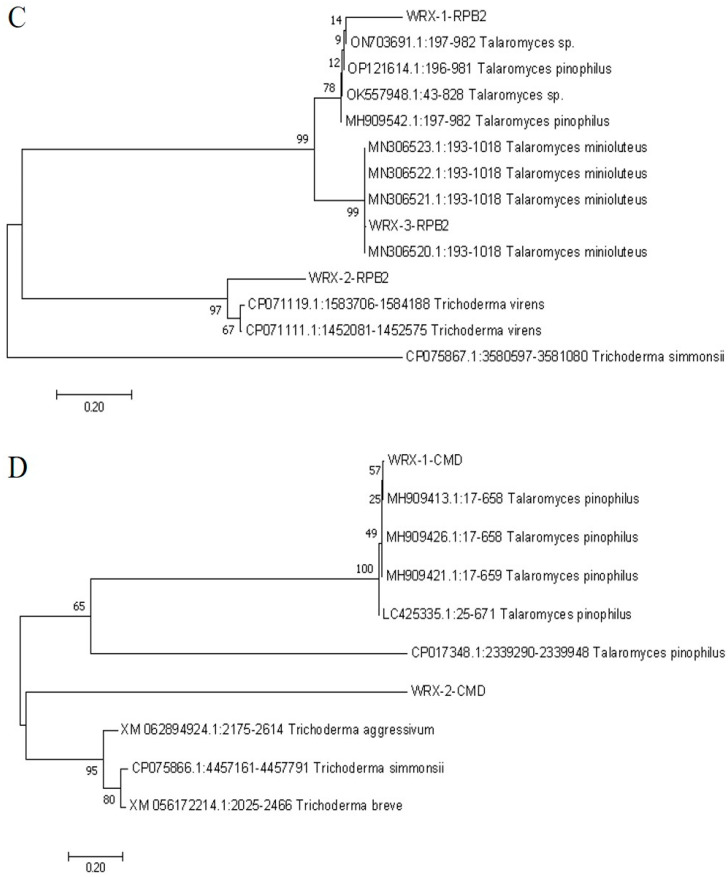
ITS, Bt, RPB2, and CMD phylogenetic trees based on the adjacency sequence method (**A**) ITS, (**B**) BT, (**C**) RBP2, (**D**) CMD.

The amplified sequences were sequenced and blasted at NCBI (https://www.ncbi.nlm.nih.gov/ (accessed 26 December 2023)). The sequences with high homology (greater than 95.00%) with isolated strains were selected. The MEGA7.0.26 (7170509-x86_64) software was used to construct ITS, Bt, RPB2, and CMD phylogenetic trees based on the adjacency sequence method (Figure 4).

The PCR amplification and phylogenetic tree analysis using ITS, BT, CMD, and RPB2 with 24 primers showed consistent results (Figure 4). WRX-1 and *T. pinophilus* (MH793130.1) were located in a branch on the phylogenetic tree with 100% homology. WRX-2 with *T. simmonsi* is (CP075867.1:3580597-3581080, CP075867.1:2245416-2245774) in the phylogenetic tree in a branch, with homology of 94%. WRX-3 shares a branch with *T. minioluteus* (MN311444.1:136-733) on the phylogenetic tree with 100% homology. However, WRX-3 is not detected for CMD. Based on the above analysis and morphological identification, WRX-1 was identified as *T. pinophilus*, WRX-2 as *T. simmonsi*, and WRX-3 as *T. minioluteus*.

### 3.3. Verification of the Pathogenicity of the Three Isolates

**Figure 5:**
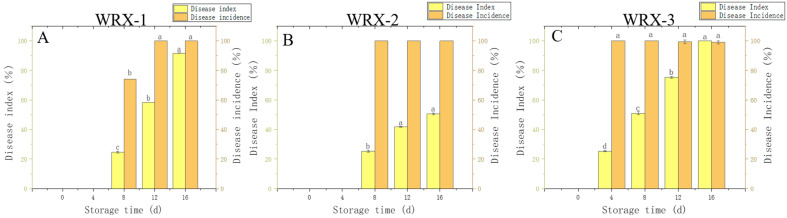
Incidences and disease indices of WRX-1 (**A**), WRX-2 (**B**), and WRX-3 (**C**). Vertical lines indicate standard error (±SE); different letters indicate statistically significant differences in variables (*p* < 0.05).

The results of the pathogenic analyses of these three isolates showed that the disease index of onions on the 16th day of storage was almost 100.00% after inoculation with those three pathogenic strains. After 16 days of storage, the incidences of WRX-1, WRX-2, and WRX-3 were 91.6%, 50.0%, and 100.00%, respectively (Figure 5). The incidence of WRX-3 was the highest, followed by WRX-1. Therefore, WRX-1 and WRX-3 are considered to be the main strains causing onion postharvest disease during storage.

### 3.4. Effects of Environmental Conditions on the Sporulation of Pathogens

#### 3.4.1. Temperature Significantly Affected the Growth and Sporulation of Pathogens

The optimum sporulation temperature for all three fungi was 25 °C. The fungi did not grow when the temperature was below 15 °C or above 40 °C. At 35 °C, the sporulation yield of the three fungi decreased significantly: *T. pinophilus* by 0.91 × 10⁷ CFU/mL, *T. simmonsii* by 0.77 × 10⁷ CFU/mL, and *T. minioluteus* by 0.77 × 10⁷ CFU/mL. When the temperature was 15 °C, the sporulation yield of *T. pinophilus* was only 1.83 × 10⁷ CFU/mL (Table 3).

#### 3.4.2. pH Significantly Affected the Growth and Sporulation of Pathogens

In the pH range of 7~8, all three strains could grow well. *T. minioluteus* grows well in a wide pH range and grows best under pH 8–9. Under pH 7, the spore production of *T. pinophilus* was 2.33 × 10^7^; compared to pH 6, it rose by 1.76 × 10^7^, but the spore production of *T. simmonsii* decreased by 0.6 × 10^7^ with the same change in pH. *T. pinophilus* and *T. simmonsii* are better suited to grow in slightly acidic environments but can also grow under alkaline conditions (Table 3).

#### 3.4.3. Light Significantly Affected the Growth and Sporulation of Pathogens

Light conditions had a significant effect on the growth of the three fungi. *T. pinophilus* (1.65 × 10^7^) and *T. simmonsii* (4.23 × 10^8^*)* had the largest sporulation under 24 h light conditions, followed by light/darkness alternations for 12 h, while *T. minioluteus* (9.30 × 10^7^) had the highest sporulation under 24 h total darkness conditions (Table 3).

#### 3.4.4. Carbon Source Significantly Affected the Growth and Sporulation of Pathogens

In the medium with six different carbon sources, all three strains could grow normally. *T. pinophilus* (2.35 × 10^7^) had the highest sporulation when maltose was the carbon source. *T. simmonsii* (10.59 × 10^8^) and *T. minioluteus* (9.23 × 10^7^) had the highest sporulation when sucrose was the carbon source (Table 3).

#### 3.4.5. Nitrogen Source Significantly Affected the Growth and Sporulation of Pathogens

The three strains grew on the five different nitrogen source media. For *T. pinophilus*, when peptone was used as the nitrogen source, the sporulation of pathogens was 2.87 × 10^7^; when urea was used as the nitrogen source, the sporulation of pathogens was 0. Therefore, peptone is the best nitrogen source for *T. pinophilus*. The highest sporulation rate was achieved by *T. simmonsii* (1.19 × 10^8^) in the medium with yeast extract powder as the nitrogen source (Table 3).

#### 3.4.6. Determination of Spore Germination of Pathogens Under Different Humidity Conditions

**Figure 6:**
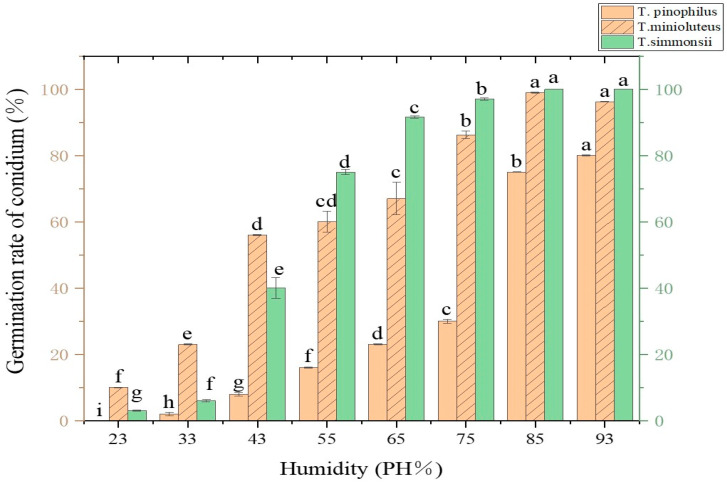
Effect of humidity on spore germination. Vertical lines indicate standard error (±SE); different letters indicate the difference of variables with statistical significance (*p* < 0.05).

The spore germination of the three fungi was greatly affected by relative humidity. When the relative humidity was lower than 23%, the spores did not germinate; when the humidity was lower than 43%, the spore germination rate was lower than 50%. The spore germination rate of *T. pinophilus* was higher than 70% when the relative humidity was higher than 85%. The spore germination rate of *T. simmonsii* was higher than 70% when the relative humidity was higher than 55%. The spore germination rate of *T. minioluteus* was higher than 70% when the relative humidity was higher than 65% (Figure 6).

### 3.5. Analysis of Onion Active Ingredients

The total ion chromatograms of volatile components from uninfected onions (control) and onions infected by WRX-1, WRX-2, and WRX-3 were obtained by analyzing the active components of the onions. The compounds were identified using the NIST 17 spectral library. The relative contents of each active substance were determined by peak area normalization. The types and relative contents of the volatile components in the onion bulbs from both the control and treatment groups are summarized in Table 4.

In the control group, 46 main volatile compounds were detected, and 40 volatile components were identified; they are primarily disulfides, such as 1-1-propenylthionyl, dipropyl trisulfide, isopropyl disulfide, and triethylsilane. In the stem rot tissues of onion scales infected with WRX-1, 44 main volatile compounds were detected, and 19 volatile components were identified, mainly disulfides, including isopropyl disulfide, (1E)-1-propenyl propyl, 2-undecanone, 2,4-octanedione, 3(2H)-furanone-2-hexyl-5-methyl, and 2-tridecanone. In the stem rot tissues of onion scales infected with WRX-2, 40 main volatile compounds were detected, with 36 volatile components identified. These were primarily disulfides, such as isopropyl disulfide, (1E)-1-propenyl propyl, 2-undecanone, 2,4-octanedione, 3(2H)-furanone-2-hexyl-5-methyl, and 2-tridecanone. In the stem rot tissues of onion scales infected with WRX-3, 41 main volatile compounds were detected, and 39 volatile components were identified; these were also primarily disulfides, including isopropyl disulfide, (1E)-1-propenyl propyl, 2-undecanone, 2,4-octanedione, 3(2H)-furanone-2-hexyl-5-methyl, and 2-tridecanone. On comparing the volatile components of uninfected onions with those infected by the three pathogens, it can be seen that 16 kinds of sulfur compounds, 4 kinds of aldehydes, and 2 kinds of alcohols were identified in uninfected onions; 19 kinds of sulfur compounds and 1 kind of alcohol were identified in onions infected with WRX-1; 16 kinds of sulfur compounds, 1 kind of aldehyde, and 1 kind of alcohol were identified in onions infected with WRX-2; and 16 kinds of sulfur compounds were identified in onions infected with WRX-3.

Although the types of sulfur-containing compounds in uninfected onions did not significantly change compared to the infected onion tissues, the relative content of these compounds was notably higher in uninfected onions. For instance, the relative content of 1-1-allyl thiopropyl disulfide in uninfected onions was 7.66%, whereas in onion bulb tissues infected with WRX-1, it was only 1.01%. Similarly, the relative content of triethylsilane in uninfected onions was 9.28%, but in onion bulb tissue infected with WRX-2, it was just 0.28%. In uninfected onion tissues, the contents of isopropyl disulfide, dipropyl triethioether, and (1E)-1-allyl propyl were 19.93%, 16.69%, and 11.57%, respectively; however, these compounds were not detected in the infected onion tissues.

**Figure 7:**
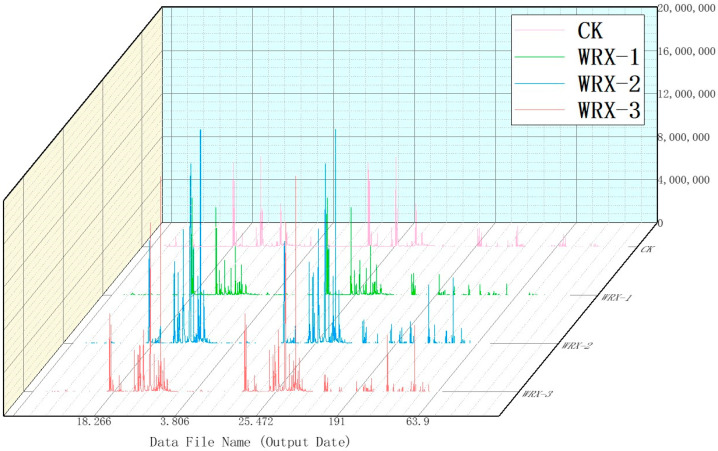
Total ion flow chromatogram of volatile components in onions.

Additionally, isopropyl disulfide, (1E)-1-propenyl propyl, 2-undecanone, 2,4-octanedione, 3(2H)-furanone-2-hexyl-5-methyl, and 2-tridecanone were detected in onions infected by all three pathogens, with no significant differences in their content among the treatments. These findings suggest that pathogen infection leads to significant changes in the types and levels of volatile compounds in onions. More secondary metabolites were produced in the onion tissues infected by the three pathogens, which affected the overall volatile components and active substances of the onions (Figure 7).

## 4. Discussion

In this study, the pathogens causing stem rot in onions were isolated, purified, and identified as *T. pinophilus*, *T. simmonsii,* and *T. minioluteus.*

*T. pinophilus* is a fungus from the genera *Cyanobacteria* and *Penicillium*, known for producing several essential bioactive metabolites, including terpenes, alkaloids, polyketides, lactones, and furan alcohols [26,27,28]. It has been widely used as an effective cellulose and waste depressant. Additionally, turquoise cyanobacteria have been shown to promote plant development in Waito-C rice and chickpea [29,30]. *T. pinophilus* also exhibits antifungal activity against *Botrytis cinerea* and *Rhizoctonia solani* [31,32]. However, studies on *T. pinophilus* in China are very limited. In this study, *T. pinophilus* was isolated and purified from onion tissues exhibiting typical stem rot symptoms.

As reported by Zhang [33], when the strain was incubated on a PDA plate at 28 °C, the colony initially appeared as white filaments, gradually turning yellow–green, and eventually producing green or dark green spores. *Talaromyces funiculosus* is commonly found in corn but can occasionally cause spoilage in other food products [34]. Zhang’s study [33] demonstrated that crude glucanase activity was purified from *T. pinophilus* through ammonium sulfate fractionation and Sepharose 6B chromatography, resulting in a 6.69 times higher recovery rate with an overall yield of 11.27%. The enzyme was identified as endoglucanase, with a molecular weight of 58 kDa, which is lower than most glucanases, and an optimal temperature and pH of 45 °C and 6.0, respectively. The enzyme was stable over a pH range of 3.0 to 10.0 and exhibited excellent thermal stability.

In recent years, research on *T. simmonsii* has primarily focused on its role in the biological control of plant diseases, with fewer reports on *T. simmonsii* as a pathogen. Yao confirmed through Koch’s postulates that *Trichoderma* spp. are the pathogens responsible for causing ear rot in maize [35]. Zhang et al. [36] isolated the fungus from soil and identified it based on rDNA-ITS fragments. Our observations showed that *T. simmonsii* appears gray–green with a yellow underside and has a velvet texture with a radial pattern underneath, which is consistent with the report by Qin [37]. Qin noted that the center of the colony was white, surrounded by a distinct circle and green areas around the central part. The aerial mycelium is distinctly radial, abundant, dense, and ranges from flocculent to cotton-like. It diffuses a light yellow pigment and has a slightly fruity odor.

*T. minioluteus* is one of the most important species of *Talaromyces* and is widely distributed worldwide. Vinas et al. isolated and purified this strain from rotten apple fruit. The fungus appears grayish-green in color, surrounded by a white fuzz, with an orange reverse side. The colonies are scattered and spherical, with spore peduncles that are curved and branched, green, and apically branched like a hand. The conidia are compact, colorless, mononuclear, spherical, concave in the middle, with a wrinkled surface, measuring (2–3.5) μm × (100–160) μm, and are produced in large numbers. Stefan Stoic [38] described the colony as brown, round, and slightly concave, with white to yellow mycelium and, in some cases, sparse to dense dark green spores on the surface of the sample. These observations suggest that the same strain isolated from different host plants can exhibit considerable variations in morphological characteristics.

In addition, different levels of relative humidity significantly impacted the spore germination of the three strains in this study. Higher relative humidity correlated with higher spore germination rates. Onions are primarily cultivated in Jiayuguan County, Gansu Province, China, where the climate features long sunshine hours, large temperature differences between day and night, and a typical Gobi Desert environment, which is favorable for onion growth. However, the storage environment differs significantly; when the humidity and temperature in storage are suitable for pathogen growth and development, postharvest diseases will worsen and cause deterioration. Consequently, the three pathogen strains are commonly employed as biocontrol agents. However, the present investigation has discerned that these bacteria are capable of inducing diseases following inoculation into onion tissues. This phenomenon may be attributed to the post-inoculation placement of the onion tissues in a sealed environment, maintained under specific temperature and humidity conditions, thereby precipitating onion pathology. Furthermore, this research represents the inaugural instance of isolating and purifying the pathogens from onion tissues. Therefore, to prevent onion stem rot, the storage environment should maintain low humidity and temperature, which will suppress the germination and growth of pathogenic fungi, thereby controlling postharvest disease [39].

The different compositions and contents of volatile components contribute to the distinct spicy flavor of onions. Sulfur-containing compounds are the main volatile components in fresh onions and are key to their characteristic flavor and active substances [40]. These sulfur-containing compounds include mono-sulfides, disulfides, trisulfides, and other related substances. They are responsible for the distinctive pungent aroma of onions [41] and possess biological activities such as lowering blood lipids, reducing blood sugar levels, and affecting hemolytic fibers.

The results of this study showed significant changes in the volatile components and their contents in onion tissues infected by *T. pinophilus*, *T. simmonsii*, and *T. minioluteus*. Notably, the content of sulfur compounds decreased significantly, while the levels of other aldehydes and alcohols increased. Vikram [42] reported that *Penicillium expansum* infection altered the types and contents of volatile compounds in McIntosh, Cortland, and Empire apples. Similarly, Encinas-Basurto et al. observed higher proportions of aliphatics (28.3%) and alkenes (4.9%) in *Botrytis cinerea*-inoculated apples, while *Monilinia*-inoculated apples contained higher proportions of esters (65.7%) and alcohols (7.8%). In the non-wounded control treatment, aromatics (10.7%) and heterocyclic compounds (9.9%) were found in normalized abundances higher than in other treatments. Encinas-Basurto et al. [43] also identified significant changes in the types and contents of volatile compounds in tomato tissues infected by *Alternaria alternata*, noting that more volatile metabolites (VMs) were released from inoculated fruits compared to control fruits. VMs such as dimethyl disulfide, 3-methyl-2-butenal, 2-methyl-1-butanol acetate, 1-butanol, 1-nitrobutane, 2-methyl-1-butenol, 4-methyl-1-pentanol, and 1-hexanol were exclusively detected in inoculated fruits. These findings indicate that pathogenic fungal infections not only lead to the deterioration of fruits and vegetables but also result in the accumulation of secondary metabolites in the affected tissues, significantly impacting the flavor, quality, and active ingredients of these produce items.

## 5. Conclusions

The pathogens causing onion stem rot during postharvest storage were identified as *Talaromyces pinophilus*, *Trichoderma simmonsii*, and *Talaromyces minioluteus*. For *T. pinophilus*, a temperature of 65 °C for 15 min was lethal, with optimal growth conditions at 30 °C, neutral pH, 24 h light, maltose as the carbon source, and peptone as the nitrogen source. For *T. simmonsii*, a temperature of 60 °C for 15 min was lethal, and the highest spore production occurred at 25 °C, neutral to slightly acidic pH, and 24 h light, with sucrose as the carbon source and yeast powder as the nitrogen source. For *T. minioluteus*, a temperature of 65 °C for 15 min was also lethal, with optimal colony growth at 25 °C, neutral to slightly acidic pH, 24 h darkness, maltose as the carbon source, and peptone as the nitrogen source. For maximum spore production, the conditions were 20 °C, neutral to slightly alkaline pH, and 24 h darkness, with sucrose as the carbon source and ammonium sulfate as the nitrogen source.

The study also found that the relative contents of sulfur compounds in onion tissues infected with WRX-1, WRX-2, and WRX-3 were significantly reduced by 16.47%, 49.35%, and 42.86%, respectively, compared to healthy and uninfected onion tissues. Furthermore, it still needs to be investigated whether mycotoxins accumulate in the onion tissues during the storage process of rotten onions and how we can affect the primary functional components of onions.

## Figures and Tables

**Table 1:** Primers and sequences for molecular biological identification of pathogenic pathogens.

Molecular Marker	Primer	Direction	Reference	Primer Sequence
Internal transcribed spacer (ITS)	ITS1	Forward	[19,20]	5′-TCCGTAGGTGAACCTGCGG-3′
	ITS4	Reverse		5′-TCCTCCGCTTATTGATATGC-3′
β-Tubulin (BenA)	Bt2a	Forward	[20]	5′-GGTAACCAAATCGGTGCTGCTTTC-3′
	Bt2b	Reverse		5′-ACCCTCAGTGTAGTGACCCTTGGC-3
Calmodulin (CaM)	AD1	Forward	[21,22]	GCCGACTCTTTGACTGAAGAGC
	AD2			GCCGATTCTTTGACCGAGGAAC
	Q1	Reverse		GCATCATGAGCTGGACGAACTC
	Q2			GCATCATGAGCTGGACGAATTC
RNA polymerase II second largest subunit (RPB2)	T1	Forward	[23]	ACTGGTAACTGGGGTGAGCA
	T2			ACGGGTAACTGGGGTGAACA
	F1	Reverse		TCACAGTGAGTCCAGGTGTG
	F2			TCGCAATGCGTCCAGGTATG

**Table 2:** Disease classification standard.

Disease Rating	Symptom
0	No disease
1	Scale disease area less than 10%
2	Scale disease area 10~30%
3	Scale disease area 30~50%
4	Scale disease area greater than 50%

**Table 3:** Effect of temperature, pH, light, and carbon and nitrogen sources on sporulation of isolated pathogens.

Culture Conditions		*Talaromyces pinophilus*	*Trichoderma simmonsii*.	*Talaromyces minioluteus*
		Spore Production (×10^7^)	Spore Production (×10^8^)	Spore Production (×10^7^)
Temperature (°C)	15	1.83 ± 0.030 ^d^	2.87 ± 0.021 ^d^	5.61 ± 0.015 ^b^
	20	2.07 ± 0.060 ^c^	4.54 ± 0.055 ^b^	6.76 ± 0.036 ^a^
	25	2.31 ± 0.015 ^b^	5.18 ± 0.000 a	4.5 ± 0.032 ^c^
	30	3.21 ± 0.010 ^a^	3.38 ± 0.003 ^c^	3.26 ± 0.012 ^d^
	35	2.30 ± 0.005 ^b^	2.61 ± 0.006 ^e^	2.74 ± 0.026 ^e^
pH	5	0.94 ± 0.006 ^b^	2.28 ± 0.329 ^a^	2.51 ± 0.015 ^g^
	6	0.66 ± 0.516 ^c^	1.88 ± 0.038 ^ab^	3.27 ± 0.057 ^f^
	7	2.33 ± 0.01 ^a^	1.82 ± 0.055 ^b^	4.04 ± 0.055 ^e^
	8	2.03 ± 0.055 ^a^	1.51 ± 0.424 ^b^	8.06 ± 0.006 ^b^
	9	1.31 ± 0.005 ^b^	0.91 ± 0.189 ^c^	8.27 ± 0.021 ^a^
	10	1.21 ± 0.01 ^b^	0.68 ± 0.010 ^cd^	5.57 ± 0.061 ^c^
	11	1.03 ± 0.006 ^b^	0.28 ± 0.002 ^d^	5.07 ± 0.061 ^d^
Light condition	24 h light	1.65 ± 0.006 ^a^	4.23 ± 0.054 ^a^	5.73 ± 0.252 ^c^
	12 h light/12 h dark	1.21 ± 0.010 ^b^	2.86 ± 0.006 ^c^	7.15 ± 0.212 ^b^
	24 dark	1.17 ± 0.210 ^b^	1.44 ± 0.055 ^b^	9.30 ± 0.042 ^a^
carbon source	glucose	0.63 ± 0.006 ^f^	3.33 ± 0.306 ^d^	1.94 ± 0.036 ^c^
	sucrose	1.73 ± 0.008 ^c^	10.59 ± 0.150 ^a^	9.23 ± 0.321 ^a^
	mannitol	1.35 ± 0.006 ^d^	3.37 ± 0.321 ^d^	3.10 ± 0.105 ^b^
	maltose	2.35 ± 0.006 ^a^	5.94 ± 0.079 ^c^	3.28 ± 0.044 ^b^
	fructose	0.77 ± 0.006 ^e^	11.83 ± 0.153 ^b^	0.47 ± 0.021 ^d^
	β-cd	1.95 ± 0.015 ^b^	3.43 ± 0.153 ^d^	0.73 ± 0.01 ^d^
nitrogen source	yeast extract	1.71 ± 0.015 ^c^	1.19 ± 0.021 ^a^	2.49 ± 0.026 ^c^
	peptone	2.87 ± 0.321 ^a^	0.52 ± 0.015 ^e^	1.37 ± 0.056 ^d^
	ammonium sulfate	1.3 ± 0.010 ^d^	0.64 ± 0.001 ^d^	3.44 ± 0.055 ^a^
	sodium nitrate	0.59 ± 0.021 ^e^	0.69 ± 0.010 ^b^	1.21 ± 0.021 ^e^
	urea	0 ± 0 ^f^	0 ± 0 ^f^	0 ± 0 ^f^
	glycine	2.42 ± 0.02 ^b^	0.86 ± 0.010 ^b^	3.07 ± 0.061 ^b^

Note: The statistical results of *T. pinophilus*, *T. simmonsi*, and *T. minioluteus*, cultured for 7 days; each data point is the average of three repeated values; different lowercase letters indicate statistically significant differences (*p* < 0.05).

**Table 4:** Analysis results of volatile components of onion by GC–MS.

Serial Number	Compound Name	CAS Number	Molecular Formula	Relative Content			
				CK	*Talaromyces pinophilus*	*Trichoderma simmonsii*	*Talaromyces minioluteus*
1	1-Propanesulfenothioic acid	137363-84-9	C_3_H_8_S_2_		0.26		
2	2,4-dimethylthiophene	638-00-6	C_6_H_8_S		0.8	0.17	0.59
3	2,3-Dithiahexane	2179-60-4	C_4_H_10_S_2_	0.08	0.13	0.22	0.06
4	methyl (1E)-1-propen-1-yl	23838-19-9	C_4_H_8_S_2_		0.07	0.11	0.05
5	Isopropyl disulfide	4253-89-8	C_6_H_14_S_2_		25.97	10.7	8
6	(1E)-1-allyl propyl	23838-21-3	C_6_H_12_S_2_		6.54	4.94	
7	Diallyl disulfide	2179-57-9	C_6_H_10_S_2_	2.25	0.49	0.23	
8	methylpropyl trisulfide	17619-36-2	C_4_H_10_S_3_	0.22	0.09	0.28	0.05
9	Propane dithioic acid	67230-81-3	C_6_H_10_S_2_	0.26	0.26		0.02
10	dicyclopropyl disulfide	68846-57-1	C_6_H_10_S_2_	0.44	0.46		
11	Butyl propyl	72437-64-0	C_7_H_16_S_2_		0.21	0.03	0.14
12	Dipropyl trisulfide	6028-61-1	C_6_H_14_S_3_		3.2	3.48	2.2
13	1E-1-propenyl-1-propyl	23838-27-9	C_6_H_12_S_3_		0.86	0.84	4.8
14	Propyl mercaptan	107-03-9	C_3_H_8_S		1.15		
15	Diallyl trisulfide	2050-87-5	C_6_H_10_S_3_		0.22		
16	3-mercapto-1,2,4-triazole	3179-31-5	C_2_H_3_N_3_S		0.16		
17	1,3,5-trithiane	116664-29-0	C_3_H_6_S_3_		0.13		
18	1-1-propenylthionyl	126876-23-1	C_7_H_14_S_3_	7.66	1.01		
19	nbutyl sulfoxide	218-511-4	C_8_H_18_OS		0.17		
20	diallyl disulfide	2179-60-4	C_4_H_10_S_2_			0.23	
21	1-allyl disulfide	122156-02-9	C_6_H_10_S_2_			0.04	0.2
22	methyl (1E)-1-propen-1-yl	23838-25-7	C_4_H_8_S_3_	0.29		0.46	0.17
23	N-(3-cyanophenyl)-2-[(5,6-dimethylthieno)]	764694-25-9	C_17_H_14_N_4_O_2_S			0.25	
24	Dipropyl trisulfide	6028-61-1	C_6_H_14_S_3_			3.48	
25	S-(2-phenoxyethyl) thioacetic acid	60359-72-0	C_10_H_12_O_2_S			0.1	
26	(1Z)-1-propenyl-1-propyl	23838-20-2	C_6_H_12_S_2_				5.36
27	2-mercapto-3,4-dimethyl-2,3-dihydrothiophene	137363-86-1	C_6_H_10_S_2_				0.82
28	(Z)-methyl-1-propenyl trisulfide	23838-25-7	C_4_H_8_S_3_				0.17
29	2-mercapto-3-4-dimethyl-2-3-dihydrothiophene	137363-86-1	C_6_H_10_S_2_				1.06
30	Di (1-propenyl) trisulfide	115321-81-8	C_6_H_10_S_3_				6.33
31	Thiopropanal S-oxide	32157-29-2	C_3_H_6_OS	0.46			
32	isopropyldisulfide	4253-89-8	C_6_H_14_S_2_	19.93			
33	Trans-diallyl disulfide	23838-23-5	C_6_H_10_S_2_	0.1			
34	Methylallyl trisulfide	34135-85-8	C_4_H_8_S_3_	0.24			
35	3-butenyl isothiocyanate	3386-97-8	C_5_H_7_NS	1.85			
36	Dipropyl trisulfide	6028-61-1	C_6_H_14_S_3_	16.69			
37	Diallyl trisulfide	2050-87-5	C_6_H_10_S_3_	1.5			
38	1-methiopropenyl-2-propenyl disulfide	126876-22-0	C_6_H_12_O_3_	0.3			
39	1-propyl sulfide	629-19-6	C_6_H_14_S_2_	2.91			
40	glutaraldehyde	111-30-8	C_5_H_8_O_2_		0.15		
41	Hexamethylcyclotrisiloxane	541-05-9	C_6_H_18_O_3_Si_3_	0.23	0.57		
42	octamethylcyclotetrasiloxane	556-67-2	C_8_H_24_O_4_Si_4_		0.22		
43	Cyclocarboxypropyl oleic acid	53980-88-4	C_21_H_36_O_4_		0.13		
44	2-nononone	821-55-6	C_9_H_18_O		0.27		
45	3, 4-trimethylsilanoxy phenethylamine	55429-13-5	C_24_H_34_F_5_NO_3_Si_3_		0.23		
46	Methyl nonyl ketone	112-12-9	C_11_H_22_O		16.59	4.88	2.36
47	2-tridecyl alcohol	1653-31-2	C_13_H_28_O		1.61		
48	2, 4-octanedione	14090-87-0	C_8_H_14_O_2_		7.2	1.13	5.07
49	N-ethyl-n-nitroso amylamine	25413-63-2	C_7_H_16_N_2_O		1.76		
50	1-methylhexyl acetate	5921-82-4	C_9_H_18_O_2_		0.94		
51	2-hexyl-5-methylfuran-3-ketone	33922-66-6	C_11_H_18_O_2_		4.1	28.9	33.87
52	1-methyl-5-methylene-8-1-methylethylene-1,6-cyclodecadiene	23986-74-5	C_15_H_24_		0.23		
53	2-tridecanone	593-08-8	C_13_H_26_O		4.59	3.38	1.44
54	2-ethyl-1-octene	51655-64-2	C_10_H_20_		0.2		
55	6-acetoxytropine	85644-59-3	C_10_H_17_NO_3_		1.24		
56	2, 4-tridecanedione	25276-80-6	C_13_H_24_O_2_		3.05	5.32	2.79
57	2-nonadecanone	629-66-3	C_19_H_38_O		0.52	0.51	
58	3-(2-methyl-1,3-dioxopentyclo-2-yl) propane-1-amine	66442-97-5	C_7_H_15_NO_2_		0.26		
59	5-methyl-2-octylfuran-3-ketone	57877-72-2	C_13_H_22_O_2_		3.72		
60	dihydroactiniolactone	17092-92-1	C_11_H_16_O_2_		0.12		
61	Tetradecane, 1,2-epoxy	3234-28-4	C_14_H_28_O		1.86		
62	2-(3-chloropropyl)-1,3-dioxane	16686-11-6	C_6_H_11_ClO_2_		0.89	1.2	
63	Cis-9-tetradecenol	35153-15-2	C_14_H_28_O		0.37		
64	4-n-heptoxyaniline formaldehyde	27893-41-0	C_14_H_20_O_2_			0.14	
65	1, 8-diazobicyclic [5.4.0] undeca-7-ene	6674-22-2	C_9_H_16_N_2_			0.81	
66	Triethylsilane	617-86-7	C_6_H_16_Si	9.28		0.28	
67	2-oxazolidinone	497-25-6	C_3_H_5_NO_2_			0.26	
68	2-triedecyl alcohol	1653-31-2	C_13_H_28_O			0.7	
69	4-methyl-4-(2,3-dimethyl-2-cyclopentenyl) pentylaldehyde	60714-25-2	C_13_H_22_O			2.47	1.31
70	12-(BOC-amino) dodecanoic acid	18934-81-1	C_17_H_33_NO_4_			0.28	
71	Nonadiol diacetate	1322-17-4	C_11_H_22_O_3_			0.14	
72	N-ethyl-n-nitroso amylamine	25413-63-2	C_7_H_16_N_2_O			0.48	
73	cycloundecanone	878-13-7	C_11_H_20_O			0.25	
74	Benzyl benzoate	120-51-4	C_14_H_12_O_2_			2.22	2.643
75	5-methyl-2-octylfuran-3-ketone	57877-72-2	C_13_H_22_O_2_			0.1	
76	Benzyl salicylate	118-58-1	C_14_H_12_O_3_			0.37	0.82
77	2-ethyl-5-methylfuran	1703-52-2	C_7_H_10_O				0.42
78	N-nonanoic acid	112-05-0	C_9_H_18_O_2_				0.78
79	2,4-pentanedione	53759-23-2	C_15_H_28_O_2_				0.5
80	Allyl stearate	6289-31-2	C_21_H_40_O_2_				0.13
81	N-ethyl-n-nitroso amylamine	25413-63-2	C_7_H_16_N_2_O				0.58
82	Beta-caryophyllene	87-44-5	C_15_H_24_				0.12
83	4-hexyl-2,5-dioxofuran-3-acetic acid	39212-21-0	C_12_H_16_O_5_				0.12
84	5,6,7,7A-tetrahydro-4,7,7 a-trimethyl-2 (4H)-benzofuranone	17092-92-1	C_11_H_16_O_2_				0.17
85	1-hexyl naphthalene	2876-53-1	C_16_H_20_				1.15
86	Pentafluorobenzyl n-caprylate	21635-03-0	C_15_H_17_F_5_O_2_				0.11
87	9-borobicyclic [3.3.1] nonane	280-64-8	C_13_H_25_BO				0.46
88	2-pentadecanone	2345-28-0	C_15_H_30_O				0.45
89	(R)-2-tert-butyl-6-methyl-1,3-dioxin-4-one	107289-20-3	C_9_H_14_O_3_				1.73
90	4H-pyrano-4-one,2,3-dihydro6-2-methylpropyl	243118-18-5	C_25_H_44_O_2_				0.62
91	acetaldehyde	200-836-8	CH_3_CHO	0.6			
92	2-ethyl butenal	19780-25-7	C_6_H_10_O	2.67			
93	m-xylene	108-38-3	C_8_H_10_	0.14			
94	2,4-dimethylthiophene	638-00-6	C_8_H_10_	0.22			
95	styrene	100-42-5	C_6_H_8_S	0.12			
96	Butyl acrylate	141-32-5	C_7_H_12_O_2_	0.23			
97	3,4-dimethylthiophene	175202-55-8	C_10_H_8_O_4_S_2_	1.61			
98	4-methylaminobenzoic acid	10541-83-0	C_8_H_9_NO_2_	0.23			
99	octamethylcyclotetrasiloxane	556-67-2	(CH_3_)_8_Si_4_O_4_	0.25			
100	2-ethylhexanol	104-76-7	C_8_HO	0.14			
101	trans-2-octenal	2548-87-0	C_8_H_14_O	0.15			
102	(1E)-1-allyl propyl	104-76-7	C_8_H_18_O	11.57			
103	((S)-(-)-2-hydroxy-3,3-dimethylbutyric acid	2511-00-4	C_11_H_20_O_2_	0.64			
104	Capric aldehyde	68846-57-1	C_6_H_10_S_2_	0.12			
105	Ethyl thiocyanate	91-20-3	C_10_H_8_	0.78			
106	2-hexyl-5-methylfuran-3-ketone	2050-87-5	C_6_H_10_S_3_	0.5			
107	oxazolidin-2-ketone	497-25-6	C_3_H_5_NO_2_	0.61			
108	Tetra-(trimethylsilanoxy) silicon	3555-47-3	C_12_H_36_O_4_Si_5_	0.11			
109	Ethyl thiocyanate	542-90-5	C_3_H_5_NS	0.42			
110	Cyclopentane carbohydrazide	3400-7-5	C_6_H_12_N_2_O	1.14			
111	1,8-bis-trimethylsiloxy-octane	16654-42-5	C_14_H_34_O_2_Si_2_	1.09			
112	dodecamethyldihydrohexasiloxane	995-82-4	C_12_H_38_O_5_Si_6_	0.24			

## Data Availability

The data presented in this study are available in this article.
